# Genome-scale CRISPR screens are efficient in non-homologous end-joining deficient cells

**DOI:** 10.1038/s41598-019-52078-9

**Published:** 2019-10-31

**Authors:** Joana Ferreira da Silva, Sejla Salic, Marc Wiedner, Paul Datlinger, Patrick Essletzbichler, Alexander Hanzl, Giulio Superti-Furga, Christoph Bock, Georg Winter, Joanna I. Loizou

**Affiliations:** CeMM Research Center for Molecular Medicine of the Austrian Academy of Sciences, Lazarettgasse 14, AKH BT 25.3, 1090 Vienna, Austria

**Keywords:** Double-strand DNA breaks, Genomic engineering

## Abstract

The mutagenic repair of Cas9 generated breaks is thought to predominantly rely on non-homologous end-joining (NHEJ), leading to insertions and deletions within DNA that culminate in gene knock-out (KO). In this study, by taking focused as well as genome-wide approaches, we show that this pathway is dispensable for the repair of such lesions. Genetic ablation of NHEJ is fully compensated for by alternative end joining (alt-EJ), in a POLQ-dependent manner, resulting in a distinct repair signature with larger deletions that may be exploited for large-scale genome editing. Moreover, we show that cells deficient for both NHEJ and alt-EJ were still able to repair CRISPR-mediated DNA double-strand breaks, highlighting how little is yet known about the mechanisms of CRISPR-based genome editing.

## Introduction

CRISPR (Clustered Regularly Interspaced Short Palindromic Repeats) - Cas9 -mediated gene editing has become a powerful approach for efficient genome editing in eukaryotic cells, where it is used to either generate loss-of-function alleles or introduce precise alterations^1–3^. The protein Cas9, together with an engineered single-guide RNA (sgRNA), forms a complex that directs the cleavage of a specific locus, by introducing a DNA double-strand break (DSB) at the DNA sequence complementary to the 23 bp protospacer-PAM (5′-NGG protospacer adjacent motif) sequence^4–6^. In human cells, DSBs are mostly repaired by the error-prone non-homologous end-joining (NHEJ) pathway that induces insertions and deletions (indels), hence disrupting gene function. In contrast, the less efficient homology directed repair (HDR) pathway makes use of a provided DNA template hence allowing for the generation of desired alterations^7–9^.

Despite the widespread use of CRISPR-Cas9 genome editing, there is still a lack of understanding about the DNA repair pathways that resolve Cas9-mediated cleavage. Supported by studies based on pharmacologic inhibition, it is widely accepted that NHEJ is the major DNA repair pathway that deals with Cas9 lesions^10–12^. However, confounders such as incomplete inhibition, off-target effects and dominant-negative patterns can skew the results of such studies, prompting us to develop genetic tools to investigate the mutagenic repair of Cas9 generated DNA breaks, using isogenic cell line models fully deficient in NHEJ. Surprisingly, our results show that NHEJ is dispensable for the repair of Cas9-induced breaks both at specific loci and using genome-scale CRISPR approaches. Moreover, we observed a differential indel signature with larger deletions in the absence of NHEJ, as well as residual editing in cells deficient for both NHEJ and alt-EJ, suggesting the existence of an alternative mechanism for the repair of Cas9-generated breaks.

## Results

**Figure 1:**
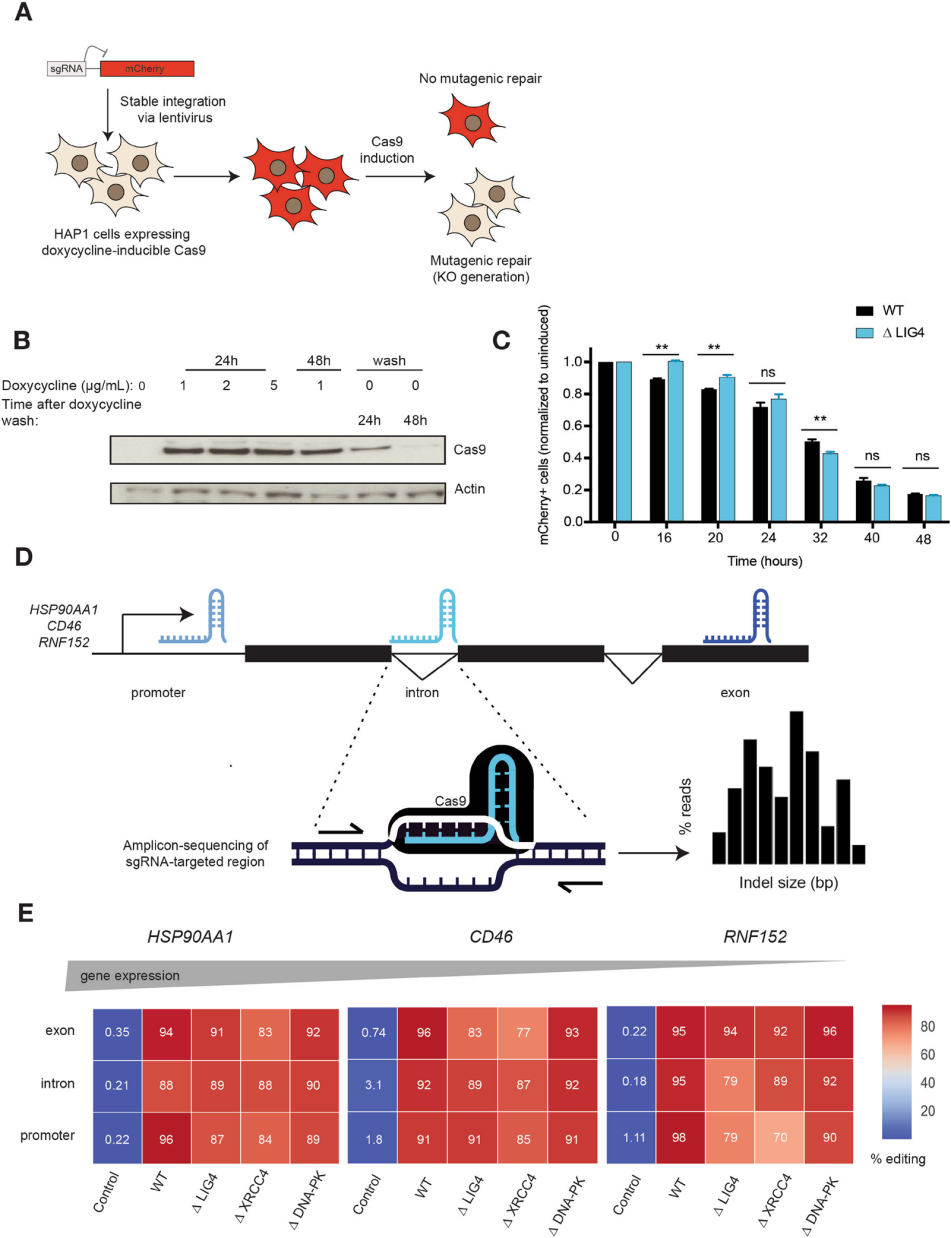
Mutagenic repair of CRISPR-Cas9-mediated DNA breaks is efficient in the absence of non-homologous end-joining. (**A**) Scheme of the cellular assay to determine the kinetics of indel generation, within the mCherry site required for fluorescence. Cells where transduced with a doxycycline-inducible Cas9-GFP and a mCherry plasmid, coupled with a sgRNA targeting the mCherry fluorescence site. Following Cas9-induction, the loss of mCherry fluorescence was used as a readout of mutagenic repair. (**B**) Immunoblot for Cas9 and ß-actin in HAP1 cells expressing doxycycline-inducible Cas9 tagged with GFP, with or without doxycycline treatment, as indicated. Figure represents cropped parts of the same gel (entire gel can be found in Supplementary Fig. 1C) (**C**). Kinetics of indel generation within the mCherry locus (measured by gating on GFP-positive cells) after Cas9-induction with doxycycline, at the indicated time points. Each time point was normalized to the uninduced (0 h) time point. The assay was performed in WT and ∆LIG4 HAP1 cells (n = 3). Statistical significance was calculated by Student’s t-test. ns = not significant, **p-value ≤ 0.01. (**D**) Scheme of the cellular assay used to measure Cas9-induced indel formation in differentially expressed genes (*HSP90AA1*, *CD46* and *RNF152*) within different genomic regions (promoters, exons and introns). Cells were transfected with Cas9 and the respective sgRNAs following which the targeted regions were PCR-amplified. Amplicon sequencing was used to determine the efficiency of editing, as well as the distribution of indel profiles. (**E**) Percentage of edited reads following Cas9 activity at promoters, exons and introns within *HSP90AA1*, *CD46* and *RNF152* in wild-type (WT) cells and knock-out cells for the NHEJ factors LIG4, XRCC4 and DNA-PKcs (*∆*LIG4, *∆*XRCC4 and *∆*DNA*-PK*, respectively).

In order to address the NHEJ dependency of mutagenic repair of Cas9-breaks, a NHEJ-deficient cell line was generated in the human HAP1 cell line, by knocking-out DNA Ligase IV (LIG4), an essential factor for the ligation of the two DNA ends^13^ (Supplementary Fig. 1A). In line with the function of NHEJ, *∆*LIG4 cells were hypersensitive to the DNA DSB-inducing agents neocarcinostatin (NCS), doxorubicin and etoposide^14^, but not to the alkylating agent methyl methanesulfonate (MMS), providing a specific phenotypic confirmation of NHEJ abrogation in this cell line (Supplementary Fig. 1B). To investigate the role of LIG4 in the repair of Cas9 generated breaks, we developed a cellular assay to measure the kinetics of genomic disruption (Fig. 1A). This consisted of expressing GFP tagged doxycycline-inducible Cas9^15^, together with a construct expressing mCherry with a sgRNA targeting the mCherry site required for fluorescence. To ensure rapid turnover of the mCherry protein, its sequence was modified to consist of a PEST sequence, hence reducing its intracellular half-life^16^. As confirmed by immunoblotting, Cas9 expression was achieved 24 hours after doxycycline treatment (Fig. 1B and Supplementary Fig. 1C), generating a DSB within the mCherry sequence that was subsequently repaired in an error-prone manner, leading to loss of fluorescence (Supplementary Fig. 1D). This system was used to assess error-prone repair leading to indel generation in wild-type (WT) and *∆*LIG4 cells, of which the later lack functional NHEJ. These results unexpectedly revealed that mutagenic repair occurs with equal efficiency in NHEJ abrogated cells as in WT cells, with 50–60% editing at 32 hours and 80% editing at 48 hours after Cas9 induction (Fig. 1C).

Following the observation that NHEJ is dispensable for Cas9-mediated editing of an exogenous locus, we designed a strategy to assess editing of endogenous loci, by testing different genomic regions (promoters, introns and exons) across genes selected to range in expression levels in the human HAP1 cell line^17^ (*HSP90AA1*, *CD46* and *RNF152*) (Fig. 1D). Upon genomic amplification of the edited region, sequencing was used to determine editing efficiency and frequency of indel size generated in the targeted loci (Fig. 1D). Moreover, we extended our investigations to include other NHEJ genes by knocking out the core component X-ray repair cross-complementing protein 4 (XRCC4) and the signaling kinase DNA-PKcs (*∆*XRCC4 and *∆*DNA-PK, respectively) (Supplementary Fig. 1E). We phenotypically confirmed that these cell lines were defective in NHEJ, by assessing their hypersensitivity to DSB-inducing agents (Supplementary Fig. 1F). Although all NHEJ deficient cell lines were exquisitely sensitive to the tested DNA DSB-inducing agents, amplicon sequencing of the Cas9 targeted regions revealed that editing was comparable to WT cells, ranging from 70–98% across all genomic regions tested, regardless of gene expression (Fig. 1E).

**Figure 2:**
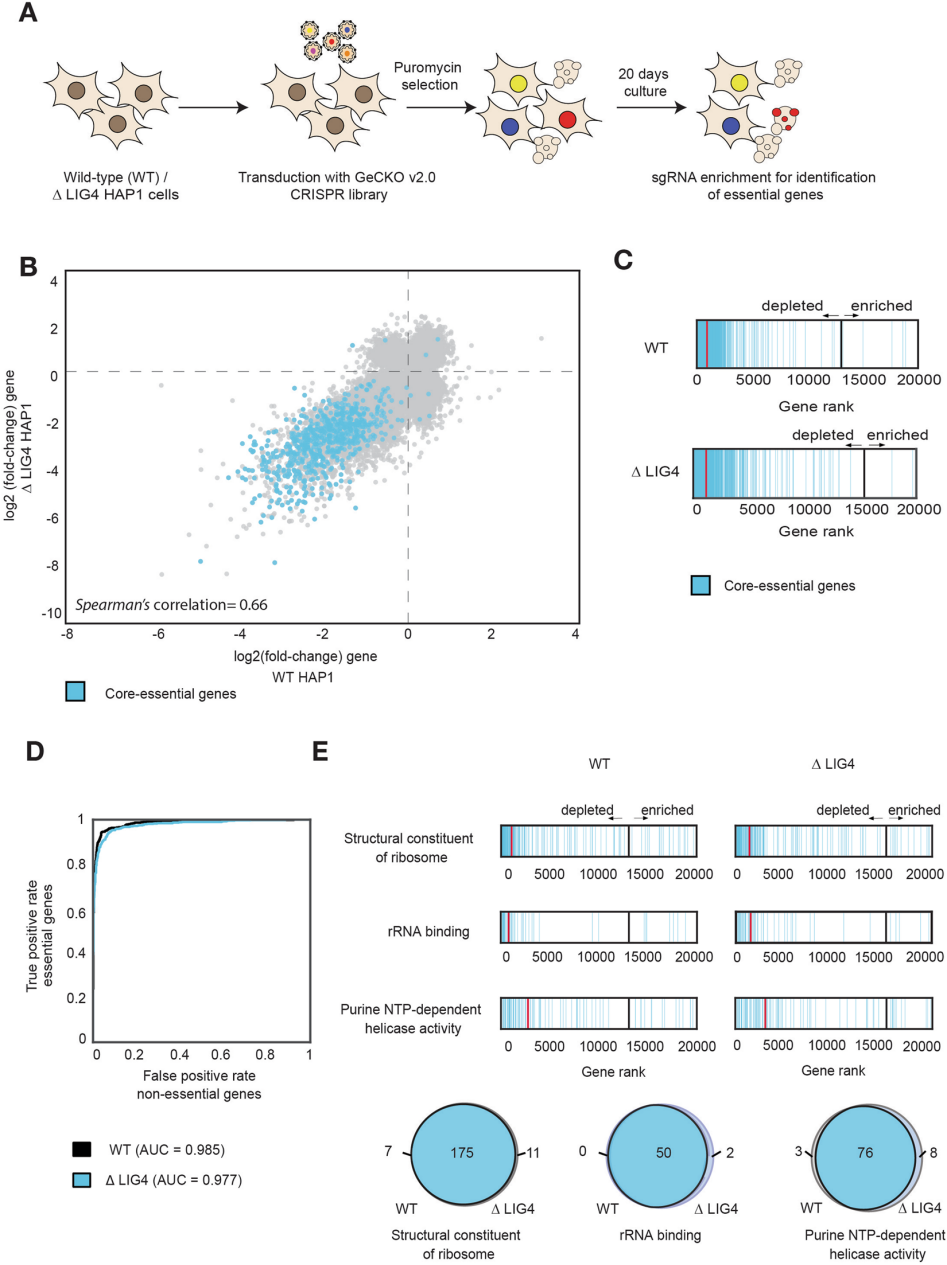
Genome-wide CRISPR-Cas9 knockout screens for global gene disruption are efficient in the absence of non-homologous end-joining. (**A**) Schematic overview of the CRISPR screens. HAP1 WT and *∆*LIG4 cells were infected at a low MOI (0.3) with the GeCKO v2.0 genome-wide CRISPR knockout library. After a period of puromycin selection, cells were kept in culture for 20 days, allowing essential genes to drop out of the population. Cells were harvested and sgRNAs were sequenced to determine relative abundances. (**B**) Scatter plot representing the log_2_(fold-change) enrichment of each gene, after culturing WT or ∆LIG4 cells transduced with the GeCKO v2.0 CRISPR library for 20 days (see *Methods* for details on the calculation of gene fold-change enrichment). Blue colored nodes represent core essential genes. Data shown for three independent experiments (n = 3). *Spearman’s* correlation (0.66) between WT and ∆LIG4 screens is depicted. (**C**) Density plot representing the position of core essential genes in the gene rank, based on log_2_(fold-change). Red lines represent the median log_2_(fold-change) of the depicted genes. Black lines represent the threshold between depleted and enriched genes. Data shown for 3 independent experiments (n = 3). (**D**) Receiver operating characteristic (ROC) analysis of depleted genes in WT and ∆LIG4 cells. False positive rates are calculated for non-essential genes and plotted against true positive rates for essential genes. Area under the curve (AUC) for each ROC curve is represented. Data shown for 3 independent experiments (n = 3). (**E**) Density plot representing the gene rank position of genes annotated for the top three enriched GO terms in the core ‘essentialome’, based on their log_2_(fold-change). Red lines represent the median log2(fold-change) of the depicted genes. Black lines represent the threshold between depleted and enriched genes. Venn diagrams represent the intersection of depleted genes for the annotated GO terms in WT and ∆LIG4 cells. Data shown for 3 independent experiments (n = 3).

So as not to limit our investigations to a single locus, we next performed genome-wide loss-of-function CRISPR-Cas9 screens, using the GeCKO v2.0 library that targets 19,052 genes with 122,417 sgRNAs^18,19^ in both WT and ∆LIG4 cells. This library allows the generation of functional null alleles at endogenous loci, in a highly multiplexed fashion, via comparative measurements of drop-outs of sgRNAs targeting 683 genes that were recently shown to be pan-essential^20^. Thus, if NHEJ would be required for CRISPR-Cas9 mediated disruption, we would expect for LIG4 deficiency to prevent the identification of essential genes. To allow for depletion of sgRNAs targeting essential genes, we analyzed sgRNA representation 20 days after puromycin selection, in both WT and LIG4 deficient backgrounds (Fig. 2A). Next-generation sequencing (NGS) was used to assess sgRNA abundance (Supplementary Table S1) and the fold-change of each gene was calculated by comparing to the sequenced library for three biological replicates (Supplementary Fig. 2A, Supplementary Table S2). This led to a *Spearman’s* correlation of 0.66 between gene enrichment in WT and *∆*LIG4 cells (Fig. 2B). Importantly, genes annotated as core essential^20^ were depleted similarly in both WT and *∆*LIG4 cells (Fig. 2B,C and Supplementary Fig. 2B). Furthermore, screens performed in both genetic backgrounds distinguish essential and non-essential^21^ genes with equal efficiency (Fig. 2D). Gene ontology (GO) analysis of core essential genes^20^ (Supplementary Fig. 2C) revealed an enrichment of essential fundamental molecular processes, including the constitution of ribosomes, rRNA binding and purine NTP-dependent helicase activity. Genes annotated for the top three enriched GO terms of the ‘essentialome’ were found depleted in both WT and *∆*LIG4 cells, with a high intersection between the genetic backgrounds (Fig. 2E). In summary, comparative identification of core essential genes using an unbiased, genome-wide approach revealed that mutagenic repair of Cas9-generated breaks can be efficiently achieved in the absence of NHEJ.

**Figure 3:**
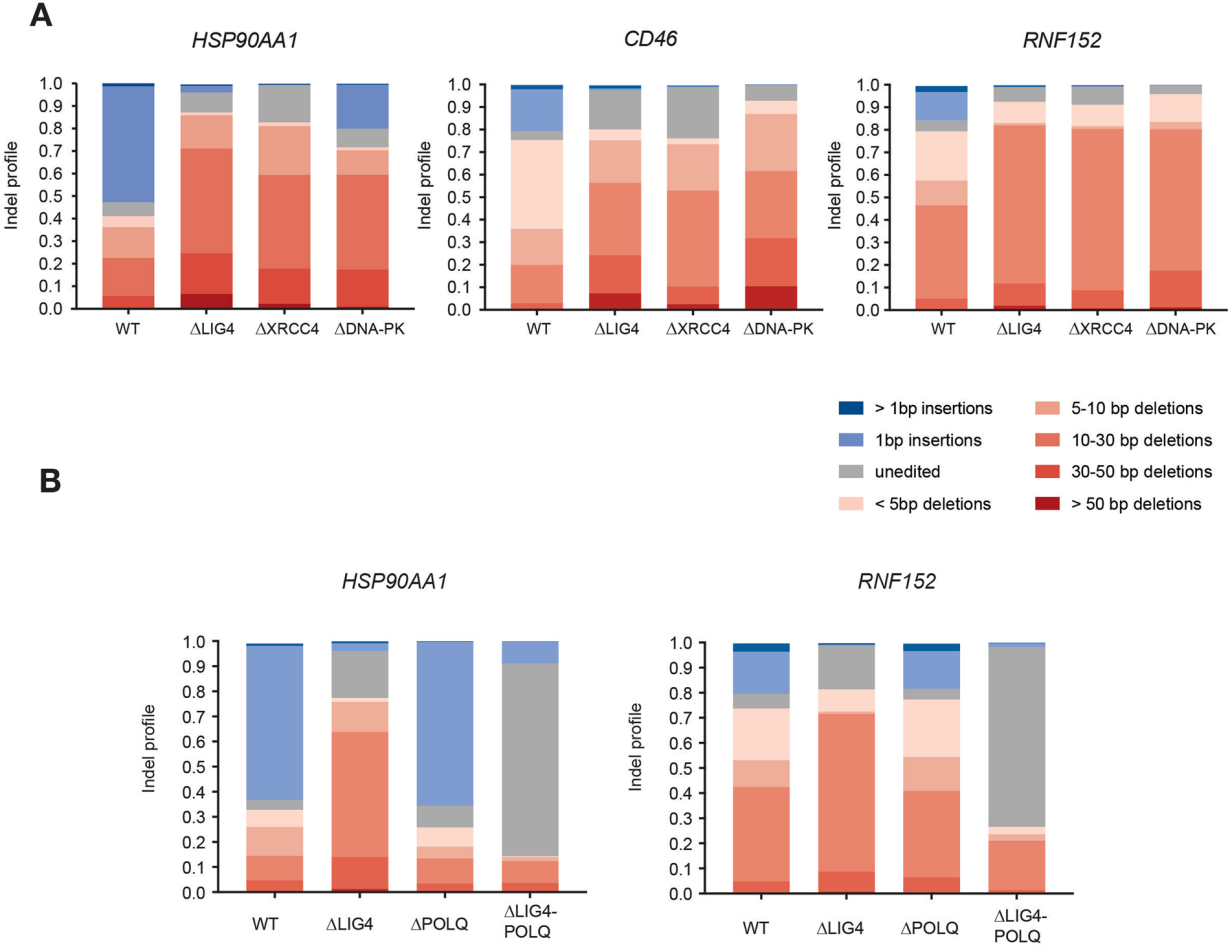
Genetic dissection of DNA repair pathway contribution to mutagenic repair of Cas9 generated lesions. (**A**) HAP1 WT, ∆LIG4, ∆XRCC4 and ∆DNA-PK cells were transfected with Cas9 and sgRNAs targeting exonic regions of 3 different genes (*HSP90AA1*, *CD46* and *RNF152*). After selection, genomic DNA was extracted and sgRNA-targeted regions were PCR-amplified. Amplicon sequencing was used to determine the indel size distribution, following editing. (**B**) Indel size distribution resulting from editing of exonic regions of HSP90AA1 and RNF152 in WT, ∆LIG4, ∆POLQ and ∆LIG4/POLQ cells, following the same procedure as described in A.

It is well documented that different sgRNAs lead to specific indel outcomes, displaying a single predominant repair outcome^11,12,22^. Following this observation, and since these predictions have important applications for template-free genome editing^23^, we sought to determine whether indel signatures would be altered in the absence of NHEJ. Besides providing the possibility of manipulating the predicted outcome of a sgRNA, this approach additionally has the potential to reveal which pathway compensates for NHEJ in the mutagenic repair of Cas9-breaks. By investigating the spectrum of indels generated upon exon targeting of three distinct genes (*HSP90AA1*, *CD46* and *RNF152*), we observed a striking increase in the frequency of larger deletions in all three NHEJ deficient cell lines, in comparison to WT cells (Fig. 3A, Supplementary Table S3). For example, the sgRNA used to target *HSP90AA1* predominantly generated 1 bp insertions (>50%) in WT cells (Fig. 3A). In NHEJ deficient cell lines, the same sgRNA generated 1 bp insertions in only 19–0.1% of the editing outcomes. Instead, 10–30 bp deletions (42–47%) were the dominant mutation pattern in these genetic backgrounds. Moreover, for sgRNAs that prominently generated deletions, we observed an increase in the size of these deletions in NHEJ-abrogated cells. For the *CD46*-targeting sgRNA (Fig. 3A), deletions smaller than 5 bp in WT cells (39%) were considerably decreased in NHEJ abrogated cell lines (2.5–5.8%), giving rise to larger deletions. A similar trend was observed for the *RNF152*-targeting sgRNA (Fig. 3A) and for other sgRNAs targeting different exonic regions, introns or promoters of these genes (Supplementary Fig. 3A, Supplementary Table S3). Importantly, our study is limited to the analysis of indels < 80 bp. Even though this range covers the majority of Cas9-proximal editing events, it does not allow speculation on larger rearrangements.

The observed shift in indel size suggested the activity of a distinct DNA repair pathway that is able to fully compensate for the loss of NHEJ, leading to the mutagenic repair of Cas9-generated DNA breaks. Since alt-EJ (also known as microhomology-mediated end joining) is known to generate larger rearrangements, we hypothesized that this might be the pathway responsible for the editing observed. The first step in alt-EJ involves 5′-end resection, to expose and allow the base-pairing of flanking regions of microhomology (MH), across the border of the DSB^24^. To test if alt-EJ is the pathway active at such lesions, we generated cells lacking the proofreading-deficient A-family DNA polymerase theta (POLQ; *∆*POLQ), the polymerase that functions in alt-EJ, following MH annealing^13^ (Supplementary Fig. 3B). Additionally, a cell line defective in both NHEJ and alt-EJ was generated by knocking-out POLQ, in the previously generated *∆*LIG4 cell line (Supplementary Fig. 3B). The abrogation of alt-EJ was phenotypically confirmed in *∆*POLQ and *∆*LIG4*/*POLQ cells, by measuring their sensitivity to several DSB-inducing agents (Supplementary Fig. 3C). As expected, *∆*POLQ cells were more sensitive to DSB-inducing agents than WT cells, but more resistant than *∆*LIG4 cells, since alt-EJ is not considered to be the main pathway by which DSBs are repaired^25^. *∆*LIG4*/*POLQ cells displayed an additive sensitivity to DSB-inducing agents. Indel signature analysis of cells, transfected with sgRNAs targeting exonic regions of *HSP90AA1* and *RNF152* (Fig. 3B), showed that WT and *∆*POLQ cells have a very similar indel profile that differed from the larger deletions observed in *∆*LIG4 cells. This finding indicates that NHEJ is the default pathway that repairs Cas9-generated breaks and illustrates that the editing observed in *∆*LIG4 cells is the product of a distinct and alternative pathway. This observation was extended to the targeting of an additional exon sequence, as well as intronic and promoter regions of these genes (Supplementary Fig. 3D).

We observed that 72–77% of the analyzed reads in *∆*LIG4*/*POLQ cells corresponded to the unedited genomic sequence, indicating that mutagenic repair of Cas9-breaks was largely impaired in these double deficient cells (Fig. 3B, Supplementary Fig. 3D). This observation was further confirmed by assessing the kinetics of mCherry editing (Supplementary Fig. 3E) and indicates that alt-EJ, via POLQ, is indeed responsible for the mutagenic repair observed in NHEJ deficient cells, leading to the generation of larger indels. Surprisingly, however, in the absence of both pathways, indel generation was still achieved in 10–20% of reads (Fig. 3B and Supplementary Fig. 3D).

## Discussion

The repair of DSBs has been widely studied by using the nuclease I-*Sce*I. However, considering that the structure of DNA termini affects repair outcome, it is important to highlight that the I-*Sce*I nuclease generates a staggered cut leaving a 3′ overhang, whereas Cas9 generates blunt ends^4–6^. In line with this, NHEJ is predominantly precise when functioning on DNA breaks introduced by the I-*Sce*I nuclease^26^, but largely error-prone when functioning on blunt ends^27,28^. Moreover, the Cas9-sgRNA complex has been shown to adhere to DNA for several hours post-cutting, a phenomenon that impacts the outcome and fidelity of DSB repair^9,11^. Hence, the repair of Cas9-induced DSBs is not representative of I- *Sce*I induced breaks and therefore further research is warranted to elucidate these repair mechanisms. This is particularly relevant in light of the therapeutic potential of CRISPR-Cas9 based technologies and especially considering that error-prone pathways are being explored to correct disease-relevant mutations^23,29^.

Following the generation of a DSB, different pathways engage in its repair, with NHEJ and other end-joining pathways, such as alt-EJ and single-strand annealing (SSA), contributing to different amounts^30,31^. When NHEJ is absent, due to the lack of one key protein, the activity of other end-joining pathways becomes apparent. Alt-EJ pathways typically require larger regions of microhomology, with the POLQ-dependent alt-EJ pathway requiring between 2–20 bp of microhomology, compared to the NHEJ microhomology requirement of ≤4 bp. Alternatively, SSA requires >20 bp homology^13^. End-resection is therefore the first barrier that needs to be overcome in order to enable alt-EJ pathways to function, with NHEJ factors such as Ku70/80^32^ and the p53-binding protein 1 (53BP1)^33^, present at high concentrations, preventing this from happening. Additionally, extensive end-resection is also dependent on cell cycle, as factors that promote end-resection are more active during S and G2 phases^33^. Hence, in G1 phase, DSBs are preferentially repaired by NHEJ and even during S and G2 phases, when extensive end resection can take place, the resection machinery must still overcome the presence of NHEJ factors at DNA ends. This is well represented by the 4:1 estimated ratio of NHEJ to HDR in WT mammalian somatic cells in S/G2 phases^34^. If NHEJ is absent, alt-EJ may be favored over SSA in G1 phase, owing to the limited amount of resection that alt-EJ requires compared to SSA. However, it is still not clear what dictates the use of alt-EJ as opposed to SSA in S/G2 phases. Time can be an important factor, as the longer a DSB remains unrepaired, the more end processing can occur to favor SSA. In our study, contrary to NHEJ deficiency, alt-EJ deficiency led to an indel profile that was very similar to that observed in WT cells. This confirms the current view that NHEJ is the main pathway by which Cas9-breaks are repaired and that alt-EJ plays only a minor role. However, the high efficiency of editing in NHEJ deficient cells, together with the almost complete abrogation of mutagenic repair in *∆*LIG4/POLQ cells, indicates that alt-EJ, in a POLQ-dependent manner, can fully compensate for the absence of NHEJ. This can have important applications for improving error-free repair, as the simultaneous transient inhibition of LIG4 and POLQ might increase HDR efficiency. Additionally, our results indicate that, in the absence of both NHEJ and alt-EJ, editing is still possible albeit with reduced efficiency (10–20%). This observation suggests the possible existence of an additional DNA repair mechanism that deals with Cas9-generated lesions. We speculate that SSA might be a potential pathway for the residual repair observed in the absence of both NHEJ and alt-EJ.

Taken together, our results show that mutagenic repair of DSBs generated by Cas9 can occur efficiently in the absence of NHEJ. We draw this conclusion utilizing genetic models where NHEJ has been abrogated, as opposed to chemical inhibitors that might lead to off-target effects and incomplete inhibition^35^. While it is theoretically possible that, in the absence of NHEJ, the repair is predominantly error-free, leading to a continuous Cas9 cutting until the target site is no longer homologous to the sgRNA, we do not favour this hypothesis based on the kinetics of mCherry-editing. Here, several cycles of Cas9 cleavage and repair would result in a considerable delay in repair of NHEJ deficient cells compared to WT cells. Since we observed similar kinetics of editing between WT and *∆*LIG4 cells, coupled with the low rates of HDR in the absence of a provided repair template^36^, we conclude that NHEJ is dispensable for the efficient mutagenic repair of Cas9-breaks. This is further confirmed by the efficiency of genome-wide CRISPR loss-of-function approach in the absence of NHEJ, which indicates for the first time, and in a holistic approach, that global-gene disruption by CRISPR-Cas9 is independent of NHEJ.

In the context of CRISPR-Cas9-mediated editing, it has been described that the repair outcomes are predominantly determined by the sgRNA sequence, rather than genomic context^12^. Following this observation, several studies have shown that each sgRNA generates a preferential editing outcome, a prediction that might have important applications for template-free genome editing^23^. By amplicon sequencing of several targeted regions, we were able to confirm the individual editing biases of sgRNAs. Moreover, we observe that these outcomes can be manipulated by the abrogation of NHEJ. NHEJ deficiency led to a distinct indel profile, characterized by the absence of small insertions and the predominance of larger deletions (10–30 bp). We hypothesize that this observation may have important applications for mutagenizing non-coding regions of the genome to disrupt, for example, the binding of transcription factors. As the implementation of functional genetic screens for non-coding transcriptional regulatory elements has been hampered by the small indel size generated by NHEJ (<10 bp)^37,38^, we speculate that the larger indels produced upon inhibition of this pathway might accelerate the development of CRISPR-Cas9 approaches for the identification of active functional enhancers, in a high-throughput manner.

## Materials and Methods

### Cell lines and culture conditions

Human HAP1 cells were obtained from Horizon Discovery and were grown in Iscove’s Modified Dulbecco’s Medium (IMDM) from GIBCO^®^, containing L-Glutamine and 25 mM HEPES and supplemented with 10% Fetal Bovine Serum (FBS) and 1% Penicillin/Streptomycin (P/S). All cell lines were diploid at the time of the experiments. HEK293T cells were obtained from the CRUK Cell Facility and were used for virus production, by culturing in Dulbecco’s modified Eagle medium (DMEM) and supplemented with 10% FBS.

### Plasmids

The human GeCKO v2.0 CRISPR knockout pooled library was a gift from Feng Zhang (Addgene #1000000048). Lenti-iCas9-neomycin was a gift from Qin Yan (Addgene # 85400). For the pCROP-mCherry-PEST plasmid, CROPseq-Guide-Puro vector (Addgene #86708) was initially digested with BsiWI/MluI and the puromycin resistance was replaced with PCR-amplified mCherry. The obtained plasmid was digested using BsrGI/MluI and a gene block (IDT) containing homology overhangs and a PEST sequence was inserted via Gibson-assembly (NEB HiFi Assembly), according to the manufacturer’s protocol. The plasmid was then digested with BsmBI and a sgRNA targeting the active site of mCherry was inserted in the place of the filler.

sgRNA mCherry:

forward: 5′-CACCGTTGGAGCCGTACATGAACTG-3′

reverse: 5′-AAACCAGTTCATGTACGGCTCCAAC-3′

BsrGI/MluI gene block (IDT):

5′-TCCACCGGCGGCATGGACGAGCTGTACAAGAAGCTTAGCCATGGCTTCCCGCCGGAGGTGGAGGAGCAGGATGATGGCACGCTGCCCATGTCTTGTGCCCAGGAGAGCGGGATGGACCGTCACCCTGCAGCCTGTGCTTCTGCTAGGATCAATGTGTAGTAAACGCGTTAAGTCGACAATCAACCTCTG-3′

### CRISPR-Cas9-mediated gene editing

CRISPR-Cas9 knockouts of LIG4, DNA-PKcs, XRCC4, POLQ and LIG4/POLQ were generated in collaboration with Horizon Genomics. Sequences for sgRNAs were designed by Horizon Genomics or with the use of http://chopchop.cbu.uib.no/. Sequences of sgRNAs used were:

LIG4: 5′-AAGGTCGTTTACTTGCTGTA-3′

XRCC4: 5′-TTACTGATGGTCATTCAGCA-3′

DNA-PK: 5′-ATAGAGCTGGTACATGGGTG-3′

POLQ: 5′-GATTCGTTCTCGGGAAGCGG-3′

### Sanger sequencing

Genomic DNA was extracted using the QIAGEN DNeasy Blood & Tissue Kit, according to the manufacture’s protocol. Genomic regions around the sgRNA-targeted sequences were amplified using the following primer pairs:

LIG4-forward: 5′-GTAGTGACATTATGCAACTCAGCAG-3′

LIG4-reverse: 5′-TAGAGATGGAAAAGATGCCCTCAAA-3′

XRCC4-forward: 5′-TGAGAGGCCAGTACAGAAAACATTA-3′

XRCC4-reverse: 5′-ACCTGTGTATAAATTTGACAGCAAT-3′

DNA-PK-forward: 5′-CTGCTGACCACTGAATTAGACAAAC-3′

DNA-PK-reverse: 5′-TTGCAGCCTGTGAACTTTTACATAG-3′

POLQ-forward: 5′-AGTAGAAGCCCAATGGGGTATG-3′

POLQ-reverse: 5′-GAGGTTTGAGTTTGAAGACTGGC-3′

PCR amplification conditions were as follows: heat lid 110 °C; 94 °C 2 min; loop 35 × (94 °C 30 s; 55 °C 30 s; 68 °C 1 min) 68 °C 7 min. Frameshift mutations were confirmed using Nucleotide BLAST against the reference genome GCF_000001405.33.

### Dose-response curves

Dose-response curves for neocarzinostatin (NCS), doxorubicin, etoposide and methyl methanesulfonate (MMS) were performed in 96-well plates, by seeding 1,000 HAP1 cells per well, the day before treatment. The following day, compounds were added at twofold serial dilutions, from the highest dose (NCS: 500 mg/mL; doxorubicin: 125 nM; etoposide: 2 µM; MMS: 750 nM). Four days after treatment, cell viability was measured using Cell Titer-Glo (Promega).

### Immunoblotting and antibodies

Cell extracts were prepared in RIPA lysis buffer (NEB) supplemented with protease inhibitors (Sigma) and phosphatase inhibitors (Sigma, NEB). Immunoblots were performed using standard procedures. Protein samples were separated by sodium dodecyl sulfate-polyacrylamide gel electrophoresis (SDS-PAGE) (3–8% gradient gels, Invitrogen) and subsequently transferred onto nitrocellulose membranes. Primary antibodies for Cas9 (7A9-3A3, Cell Signaling Technology #14697) and ß-Actin (A5060, Sigma) were used at 1:1,000. Secondary antibodies were used at 1:5,000 (HRP-conjugated goat anti-mouse or anti-rabbit IgG from Jackson Immunochemicals). Immunoblots were imaged using a Curix 60 (AGFA) table-top processor.

### Kinetics of indel generation for mCherry active site

#### Virus production

HEK293T cells were seeded in 6-well plates at 200,000 cells per well and transfection was performed the following day with 0.3 µg per well of the VSG and 0.5 µg per well of the psPAX2 packaging vectors, together with 1 µg per well of either the iCas9-GFP vector, or the pCROP-mCherry-PEST vector. The Effectene Transfection Reagent (QIAGEN) was used at 20 µL per well. Supernatant containing the viral particles was harvested two- and three-days post-transfection and filtered with a 0.45 µm filter (Milipore Steriflip HV/PVDF). Viral supernatants were stored at −80 °C.

#### Generation of iCas9-GFP, pCROP-mCherry-PEST cell lines

Cells were first transduced with the iCas9-GFP vector, using a virus dilution of 1:12 in a 24 well-plate and 8 µg/mL of polybrene. Spin-infection was performed at 2,000 rpm, 30 minutes, at 30 °C. In order to enrich for Cas9-expressing cells, transduced cells were treated with doxycycline (2 µg/mL) for 24 hours and the GFP positive population was sorted, using a SH800S Cell Sorter (Sony Biotechnology). Sorted cells were kept in culture, in the absence of doxycycline, for at least one week. After this period, cells were transduced with the pCROP-mCherry-PEST plasmid, following the same spin-infection protocol, and then sorted for mCherry-positive cells.

#### Kinetics of mCherry editing

To assess the kinetics of indel generation, 20,000 cells per well were plated in triplicate in a 12-well plate. Doxycycline (2 µg/mL) was added to the medium at the indicated time points and cells were analyzed in a BD LSRFortessa flow-cytometer. mCherry fluorescence was assessed upon gating on GFP-positive cells.

### Genome-wide CRISPR-Cas9 screen

#### Virus production

The GeCKO v2.0 CRISPR library virus was produced as reported by the distributor (Addgene #1000000048) using both library A and B in a one-production step. HEK-293T cells were seeded at 40% confluency in T-225 flasks and transfected, 24 hours later, with the GeCKO library A and B, pVSVG and psPAX2 packaging plasmids, using Lipofectamine^®^ 2000 (Invitrogen, ThermoFisher Scientific), according to the manufacturer’s protocol. After 6 hours, medium was changed to DMEM (10% FBS) and after 60 hours, supernatant-containing virus was harvested and filtered through a 0.45 µm filter (Milipore Steriflip HV/PVDF).

#### Screen setup

Three biological replicates were performed for each screen. HAP1 cells were infected at a multiplicity of infection (MOI) between 0.3–0.5. For each cell line (WT and *∆*LIG4), 100 million cells were spin-infected. Day 1: 6 12-well plates were seeded with 1.5 million cells per well, supplemented with viral supernatant and IMDM (10% FBS, 1% P/S) to reach a volume of 1 mL per well. Polybrene was added at the final concentration of 8 µg/mL. Cells were spin-infected at 2,000 rpm, 37 °C, for 3 hours, pooled and transferred to 15 cm dishes. Day 2: Cells were exposed to 2 µg/mL of puromycin to select for infected cells. Day 7: Medium was replaced with IMDM (10% FBS, 1% P/S). Cells were kept in culture for 20 days after puromycin selection and split every 2–3 days to avoid confluency, re-seeding > 100 million cells each time. After this period, cells were harvested and genomic DNA was extracted using the QIAGEN Blood & Cell Culture Maxi Kit, according to the manufacturer’s protocol.

#### sgRNA amplification and sequencing

Amplification of the sgRNA sequences was performed in a two-step PCR, using PCR1- and barcoded PCR2-primers, as described by the distributor (Addgene). Primer sequences were obtained from http://genome-engineering.org/gecko/wp-content/uploads/2013/12/GeCKO-plasmid-readout-primers-July2014.xlsx. PCR1 amplified the sgRNA sequences, using 130 µg genomic DNA in 13 × 100 µL reactions per sample and GoTaq G2 DNA Polymerase (Promega). PCR1 program: Heat lid 110 °C; 94 °C 2 min; loop 20 × (94 °C 30 s; 55 °C 30 s; 68 °C 1 min) 68 °C 7 min. PCR1 reaction tubes were pooled for each sample. PCR2 added Illumina sequencing adapters by using 2 µL of input from PCR1. A test PCR with different amplification cycles was conducted and products were ran on a 0.8% agarose gel. The number of cycles for which a band with approximately 380 bp was visible, but not saturated, was selected (*n*). PCR2 was then performed following the program: Heat lid 110 °C; 94 °C 2 min; loop *n* x (94 °C 30 s; 55 °C 30 s; 68 °C 1 min) 68 °C 7 min. PCR2 product was purified by size-exclusion, using magnetic AMPure XP DNA beads (NEB), using a 1:0.45 ratio to remove fragments >1,000 bp, followed by a 1:2 ratio clean-up. Barcoded samples were pooled and sequenced using 61 base-pair single-end sequencing. Sequencing of the GeCKO plasmids (library A and B) was performed in the same way, using 200 ng of plasmid per reaction for PCR1.

#### Screen analysis

sgRNA sequences were retrieved by trimming all sequences 5′ relative to the adapter sequence (CGAAACACCG) and 20 nucleotides 3′ following this. MAGeCK^39^ was used to generate the sgRNA counts, using a pre-made index of the GeCKO v2.0 library. sgRNA counts were normalized to million counts, for each sequencing sample and averaged across the three biological replicates. Gene log_2_(fold-change) was calculated by selecting a best representative sgRNA for each gene, as following: 1) The log_2_(fold-change) of each sgRNA was calculated by comparing to the sequenced GeCKO library; 2) The average of the log_2_(fold-change) for all sgRNAs targeting the same gene was calculated. Genes with less than 3 sgRNAs were excluded from this analysis; 3) If the average was positive, it was assumed that the gene had a tendency to be enriched in the screen, in comparison to the sequenced library. Therefore, the sgRNA with the 2^nd^ highest log_2_(fold-change) was selected as the best representative for that particular gene. If the average was negative, it was assumed that the gene had a tendency to be depleted in the screen. Therefore, the sgRNA with the 2^nd^ lowest log_2_(fold-change) was selected as the best-representative sgRNA. By excluding the highest and lowest sgRNAs, we prevent the introduction of biases. Significance of the enrichment analysis (assessed by *p*-value) was calculated using MAGeCK, comparing the screens (WT and ∆LIG4) with the sequenced library.

Receiver operating characteristic (ROC) analysis of cell viability was calculated by filtering the 683 genes annotated to be core-essential^20^ and 927 genes annotated as non-essential^21^. The ability of each screen (WT and ∆LIG4) to distinguish between these essential (true positives) and non-essential genes (false positives) was assessed by plotting their ROC curves (False Positive Rate [FPR] *vs* True Positive Rate [TPR]) and calculating the respective Area Under the Curve (AUC). Values used for the ROC curve were based on the gene −log_10_(*p*-value).

Gene Ontology (GO) enrichment analysis of core-essential genes^20^ for molecular processes, was performed by extracting the GO annotations from the Gene Ontology Consortium [www.geneontology.org]. For every GO term, the fold-enrichment was computed over a background comprising the entire human genome. *p*-value was calculated by Fisher’s exact test and adjusted by *Bonferroni* correction.

### Indel analysis by next generation sequencing

Amplicons were designed to have the sgRNA target site at the center of the products. sgRNAs were designed to target different genomic regions within different genes, using http://chopchop.cbu.uib.no/. sgRNA sequences can be found in Supplementary Table S4.

HAP1 cells were seeded in 6-well plates at a confluency of 40% and transfected with the respective sgRNA and Cas9 constructs the following day, using Effectene as the transfection reagent (QIAGEN), according to the manufacturer’s protocol. Transfected cells were then selected with blasticidine (20 µg/mL) for 2 days and harvested as soon as confluent (4–7 days). Genomic DNA was extracted using the QIAGEN DNeasy 96 Blood & Tissue Kit, according to the manufacturer’s protocol. PCR reactions were set in a reaction volume of 50 µL. The DNA polymerase Q5 High Fidelity (NEB) was used to amplify 100 ng of genomic DNA, using the following program: Heat lid at 110 °C; 98 °C for 30 s; loop 35 × (98 °C for 30 s, Annealing temperature (primer dependent) for 30 s, 72 °C for 1 min); 72 °C for 2 min. Primer sequences can be found in Supplementary Table S4. PCR products were purified by a 2.0 x AMPure XP bead clean-up (NEB), measured using a Qubit HS assay (Invitrogen), and used as input for Nextera XT (Illumina cat. no 15032350) library preparations, performed according to protocols provided by the supplier. Libraries were sequenced on the Illumina MiSeq platform, using a 150-cycle v3 flow-cell with dual indexing. The machine was set to read lengths of 159 (read1) + 8(i7) + 8 (i5) bases. The analysis of the data was performed by defining two 10 bp ‘anchor’ sequences on both sides of the sgRNA, at a fixed distance of 80 bp. Reads spanning the sgRNA target site were extracted from the BAM file, via a grep operation for the pattern ‘<anchor_left>. *<anchor_right>’ on the BAM file, using the –o option to return only the matching part of the sequence. For unedited fragments, this sequence equals 10 bp anchor_ left + 30 bp + 20 bp (sgRNA) + 30 bp + 10 bp anchor_right (total of 100 bp). The size of the indels were calculated as the deviation from the unedited fragment length, summarized and plotted.

### Statistical analysis and data visualization

All simulations and visualizations used the Python programming language, version 2.0 (Python Software Foundation, https://www.python.org/).

### Ethical approval

The methods described in this manuscript were carried out in accordance with the relevant guidelines and regulations.

## Supplementary information

Supplemetary information

Table S1

Table S2

Table S3

Table S4

## Data Availability

All data generated or analyzed during this study is included in this published article and its supplementary information. Sequencing of sgRNA cassettes in the 6 genome-wide CRISPR-Cas9 screens (associated with Fig. 2 and Supplementary Fig. 2) have been deposited in the NCBI Sequence Read Archive (SRA) with the final SRA accession code: PRJNA565227.

## References

[1] Doudna JA, Charpentier E (2014). The new frontier of genome engineering with CRISPR-Cas9. Science (80-)..

[2] Sander JD, Joung JK (2014). CRISPR-Cas systems for editing, regulating and targeting genomes. Nat. Biotechnol..

[3] Hsu PD, Lander ES, Zhang F (2014). Development and Applications of CRISPR-Cas9 for Genome Engineering. Cell.

[4] Cong L (2013). Multiplex Genome Engineering Using CRISPR/Cas Systems. Science (80-)..

[5] Jinek, M. *et al*. RNA-programmed genome editing in human cells. *Elife* **2**, (2013). 10.7554/eLife.00471 PMC3557905 23386978

[6] Mali P (2013). RNA-guided human genome engineering via Cas9. Science.

[7] Hustedt N, Durocher D (2017). The control of DNA repair by the cell cycle. Nat. Cell Biol..

[8] Canny, M. D. *et al*. Inhibition of 53BP1 favors homology-dependent DNA repair and increases CRISPR–Cas9 genome-editing efficiency. *Nat*. *Biotechnol*. **36**, (2017). 10.1038/nbt.4021 PMC5762392 29176614

[9] Richardson CD, Ray GJ, DeWitt MA, Curie GL, Corn JE (2016). Enhancing homology-directed genome editing by catalytically active and inactive CRISPR-Cas9 using asymmetric donor DNA. Nat. Biotechnol..

[10] Bothmer A (2017). Characterization of the interplay between DNA repair and CRISPR/Cas9-induced DNA lesions at an endogenous locus. Nat. Commun..

[11] Brinkman, E. K. *et al*. Kinetics and Fidelity of the Repair of Cas9-Induced Double-Strand DNA Breaks. *Mol*. *Cell* **0**, (2018). 10.1016/j.molcel.2018.04.016 PMC5993873 29804829

[12] van Overbeek M (2016). DNA Repair Profiling Reveals Nonrandom Outcomes at Cas9-Mediated Breaks. Mol. Cell.

[13] Chang HHY, Pannunzio NR, Adachi N, Lieber MR (2017). Non-homologous DNA end joining and alternative pathways to double-strand break repair. Nat. Rev. Mol. Cell Biol..

[14] Adachi N, Suzuki H, Iiizumi S, Koyama H (2003). Hypersensitivity of Nonhomologous DNA End-joining Mutants to VP-16 and ICRF-193. J. Biol. Chem..

[15] Cao J (2016). An easy and efficient inducible CRISPR/Cas9 platform with improved specificity for multiple gene targeting. Nucleic Acids Res..

[16] Rogers S, Wells R, Rechsteiner M (1986). Amino acid sequences common to rapidly degraded proteins: the PEST hypothesis. Science (80-)..

[17] Brockmann M (2017). Genetic wiring maps of single-cell protein states reveal an off-switch for GPCR signalling. Nature.

[18] Shalem O (2014). Genome-Scale CRISPR-Cas9 Knockout Screening in Human Cells. Science (80-)..

[19] Sanjana NE, Shalem O, Zhang F (2014). Improved vectors and genome-wide libraries for CRISPR screening. Nat. Methods.

[20] Hart T (2017). Evaluation and Design of Genome-Wide CRISPR/SpCas9 Knockout Screens. G3 (Bethesda)..

[21] Hart T, Brown KR, Sircoulomb F, Rottapel R, Moffat J (2014). Measuring error rates in genomic perturbation screens: gold standards for human functional genomics. Mol. Syst. Biol..

[22] Allen F (2018). Predicting the mutations generated by repair of Cas9-induced double-strand breaks. Nat. Biotechnol..

[23] Shen MW (2018). Predictable and precise template-free CRISPR editing of pathogenic variants. Nature.

[24] Seol JH, Shim EY, Lee SE (2018). Microhomology-mediated end joining: Good, bad and ugly. Mutat. Res. - Fundam. Mol. Mech. Mutagen..

[25] Saito S, Maeda R, Adachi N (2017). Dual loss of human POLQ and LIG4 abolishes random integration. Nat. Commun..

[26] Guirouilh-Barbat J, Rass E, Plo I, Bertrand P, Lopez BS (2007). Defects in XRCC4 and KU80 differentially affect the joining of distal nonhomologous ends. Proc. Natl. Acad. Sci..

[27] Boulton SJ, Jackson SP (1996). Saccharomyces cerevisiae Ku70 potentiates illegitimate DNA double-strand break repair and serves as a barrier to error-prone DNA repair pathways. EMBO J..

[28] Schär P, Herrmann G, Daly G, Lindahl T (1997). A newly identified DNA ligase of Saccharomyces cerevisiae involved in RAD52-independent repair of DNA double-strand breaks. Genes Dev..

[29] Román-Rodríguez FJ (2019). NHEJ-Mediated Repair of CRISPR-Cas9-Induced DNA Breaks Efficiently Corrects Mutations in HSPCs from Patients with Fanconi Anemia. Cell Stem Cell.

[30] Ceccaldi R (2015). Homologous-recombination-deficient tumours are dependent on Polθ-mediated repair. Nature.

[31] Mateos-Gomez PA (2015). Mammalian polymerase θ promotes alternative NHEJ and suppresses recombination. Nature.

[32] Mimitou EP, Symington LS (2010). Ku prevents Exo1 and Sgs1-dependent resection of DNA ends in the absence of a functional MRX complex or Sae2. EMBO J..

[33] Escribano-Díaz C (2013). A Cell Cycle-Dependent Regulatory Circuit Composed of 53BP1-RIF1 and BRCA1-CtIP Controls DNA Repair Pathway Choice. Mol. Cell.

[34] Beucher A (2009). ATM and Artemis promote homologous recombination of radiation-induced DNA double-strand breaks in G2. EMBO J..

[35] Jiang W (2015). Differential Phosphorylation of DNA-PKcs Regulates the Interplay between End-Processing and End-Ligation during Nonhomologous End-Joining. Mol. Cell.

[36] Richardson CD (2018). CRISPR–Cas9 genome editing in human cells occurs via the Fanconi anemia pathway. Nat. Genet..

[37] Korkmaz G (2016). Functional genetic screens for enhancer elements in the human genome using CRISPR-Cas9. Nat. Biotechnol..

[38] Lopes R, Korkmaz G, Agami R (2016). Applying CRISPR–Cas9 tools to identify and characterize transcriptional enhancers. Nat. Rev. Mol. Cell Biol..

[39] Li W (2014). MAGeCK enables robust identification of essential genes from genome-scale CRISPR/Cas9 knockout screens. Genome Biol..

