# Supplementary material for: Genome-scale CRISPR screens are efficient in non-homologous end-joining deficient cells
Source: Sci Rep. 2019 Oct 31;9:15751. doi: 10.1038/s41598-019-52078-9 (PMC6823505; doi:10.1038/s41598-019-52078-9)
Supplement: Supplementary file 1 — Supplemetary information [file 41598_2019_52078_MOESM1_ESM.docx]

**Genome-scale CRISPR screens are efficient in non-homologous end-joining deficient cells**

Joana Ferreira da Silva^1^, Sejla Salic^1^, Marc Wiedner^1^, Paul Datlinger^1^, Patrick Essletzbichler^1^, Alexander Hanzl^1^, Giulio Superti-Furga^1^, Christoph Bock^1^, Georg Winter^1^, Joanna I. Loizou^1^*

**Supplementary Information**

**
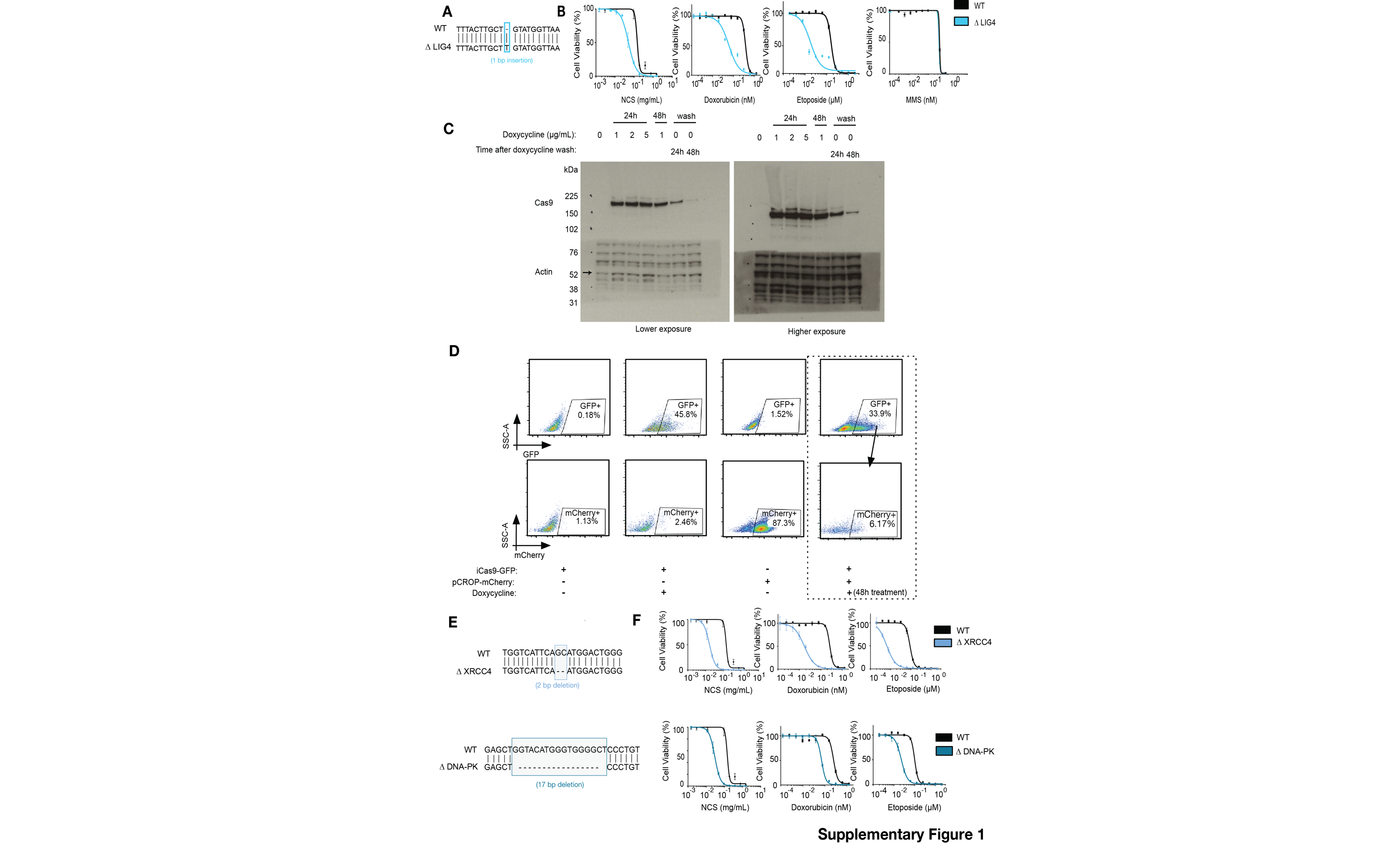
**

**Supplementary Figure 1: Generation of a relevant cellular system to assess the kinetics of Cas9-activity**. **A.** CRISPR-Cas9 generated clonal HAP1 cell line carrying a 1bp insertion in the *LIG4* gene. **B.** Dose-response curves for WT and *∆*LIG4 HAP1 cell lines to the DNA double-strand break-inducing agents neocarcinostatin (NCS), doxorubicin and etoposide and to the alkylating agent methyl methanesulfonate (MMS). Cells were treated with the indicated compounds for 3 days and viability was measured by Cell Titer Glo. **C.** Immunoblot for Cas9 and ß-actin in HAP1 cells expressing doxycycline-inducible Cas9 tagged with GFP, with or without doxycycline treatment, as indicated. Figure represents the entire gel, with different exposure times. **D.** Representative FACS-plots for WT HAP1 cells expressing the indicated integrated constructs, with or without doxycycline treatment for Cas9 induction. mCherry editing was assessed by gating GFP-positive cells. **E.** Generation of *∆*XRCC4 and *∆*DNA*-PK* clonal HAP1 cell lines, with a 2bp and a 17bp deletion, respectively. **F.** Dose response curves for WT, *∆*XRCC4 and *∆*DNA-PK HAP1 cells to the DNA double-strand break-inducing agents neocarcinostatin (NCS), doxorubicin and etoposide. Cells were treated with the indicated compounds for 3 days and viability was measured using Cell Titer Glo.

**
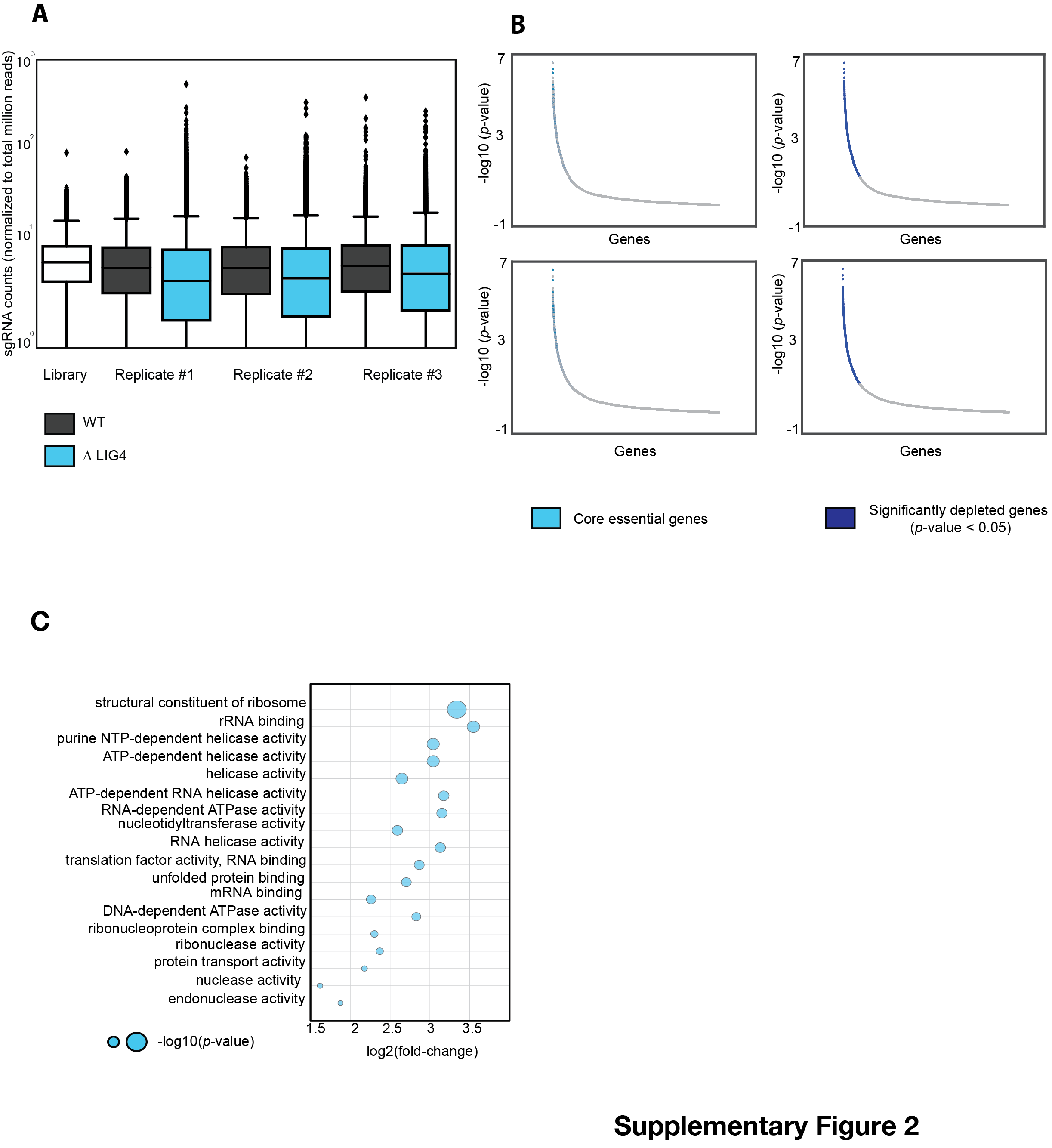
**

**Supplementary Figure 2: Genome-wide CRISPR-Cas9-KO screens identify essential genes efficiently in WT and *∆*LIG4** **cells.** **A.** Depiction of sgRNA representation, normalized to total millions of reads for the GeCKO v2.0 library (depicted as ‘Library’), as well as each biological replicate of HAP1 WT and *∆*LIG4 cells. **B.** Genes ranked by –log_10_(*p*-value), calculated with MAGeCK. Light blue colored nodes represent genes identified as core essential in the WT and *∆*LIG4 screens (*left-hand sid*e). Dark blue colored nodes represent genes considered to be significantly depleted in each screen (*p*-value < 0.05) (*right hand-side*). **C.** Gene ontology (GO) enrichment analysis for molecular processes of essential genes plotted by log_2_(fold-change). Size of the node represents statistical significance.


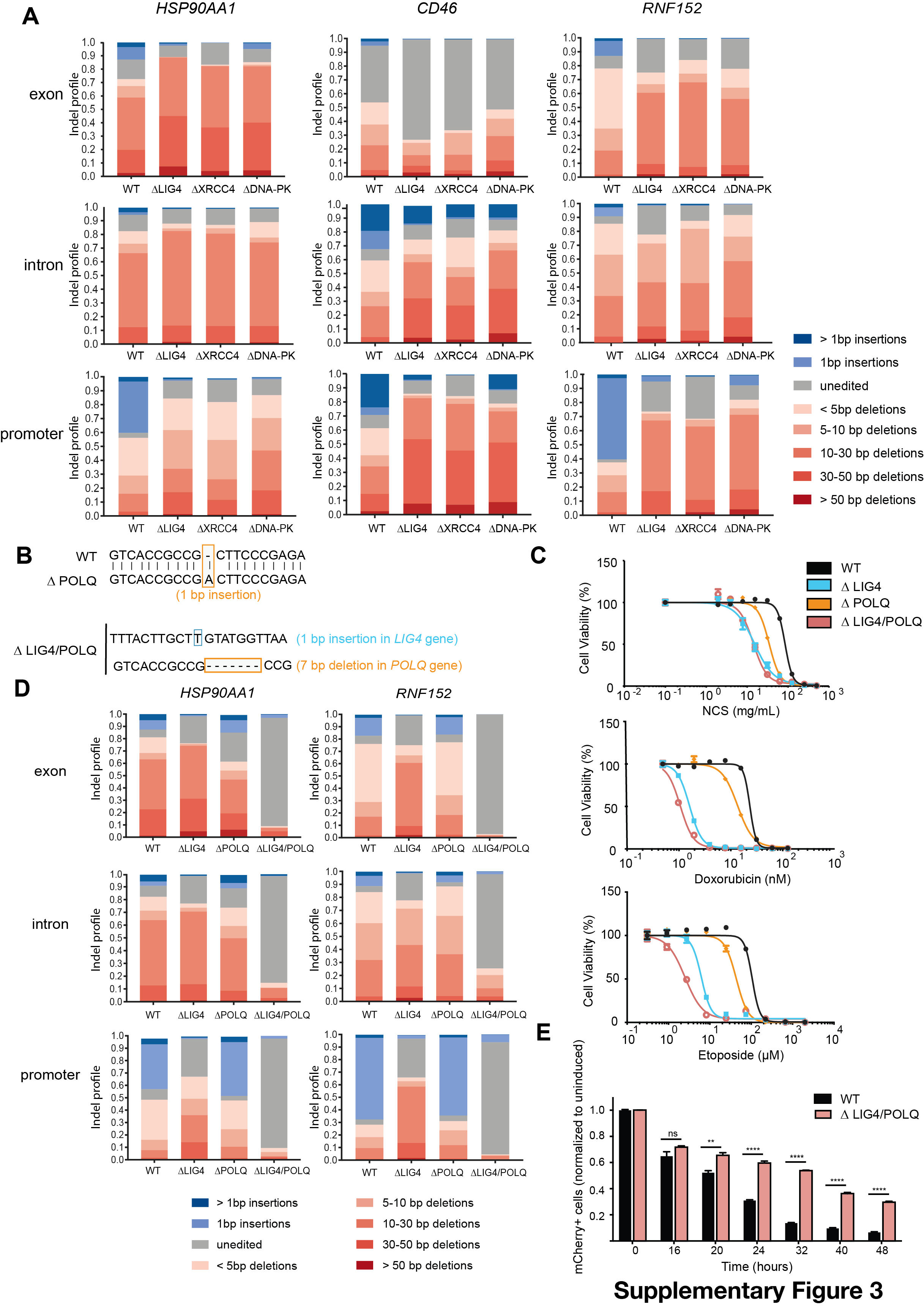


**Supplementary Figure 3: NHEJ and alt-EJ-mediated repair generate different types of indel signatures. A.** HAP1 WT, *∆*LIG4*, ∆*XRCC4 and *∆*DNA-PK cells were transfected with Cas9 and sgRNAs targeting exonic, intronic or promoter regions of 3 different genes (*HSP90AA1, CD46* and *RNF152).* After selection, genomic DNA was extracted and sgRNA-targeted regions were PCR-amplified. Amplicon sequencing was used to determine the indel size distribution, following editing. **B**. Generation of a clonal *∆*POLQ HAP1 cell line with a 1bp insertion and a clonal *∆*LIG4*/∆*POLQ double mutant HAP1 cell line (+1bp/-7bp, for LIG4 and POLQ respectively). **C.** Dose-response curves for WT, *∆*LIG4*, ∆*POLQ and *∆*LIG4/POLQ HAP1 cell lines to the DNA double-strand break-inducing agents neocarcinostatin (NCS), doxorubicin and etoposide**.** Cells were treated with the indicated compounds for 3 days and viability was measured by Cell Titer Glo**. D.** Indel size distribution in WT, ∆LIG4, ∆POLQ and ∆LIG4/POLQ HAP1 cells, following the repair of Cas9-induced breaks within exonic, intronic and promoter regions of *HSP90AA1* and *RNF152*, following the same procedure described in A. **E.** Kinetics of indel generation within the mCherry locus (measured by gating on GFP-positive cells) after Cas9-induction with doxycycline, at the indicated time points. Each time point was normalized to the uninduced (0h) time point. The assay was performed in WT and ∆LIG4/POLQ HAP1 cells (n=3). Statistical significance was calculated by the Student’s t-test. ns=not significant, ** *p*-value ≤ 0.01, **** *p*-value ≤ 0.0001.

**Supplementary Tables**

**Supplementary Table S1:** sgRNA counts normalized to total million reads for the GeCKO v2.0 sequenced library, as well as each CRIPSR screen replicate in WT and *∆*LIG4 HAP1 cells. Final log_2_(fold-change) of each sgRNA in each screen (n=3).

**Supplementary Table S2:** Log*_2_*(fold-change) of each gene for every CRISPR screen (n=3) performed in WT and *∆*LIG4 HAP1 cells, calculated based on the best representative sgRNA.

**Supplementary Table S3:** Indel-length count, measured by amplicon sequencing, for each targeted region within the genes *HSP90AA1*, *CD46* and *RNF152*.

**Supplementary Table S4:** sgRNA and primer sequences used for amplicon sequencing.
